# Models of Innate Neural Attractors and Their Applications for Neural Information Processing

**DOI:** 10.3389/fnsys.2015.00178

**Published:** 2016-01-05

**Authors:** Ksenia P. Solovyeva, Iakov M. Karandashev, Alex Zhavoronkov, Witali L. Dunin-Barkowski

**Affiliations:** ^1^Department of Neuroinformatics, Center for Optical Neural Technologies, Scientific Research Institute for System Analysis, Russian Academy of SciencesMoscow, Russia; ^2^Laboratory of Functional Materials and Devices for Nanoelectronics, Department of Nanometrology and Nanomaterials, Moscow Institute of Physics and TechnologyDolgoprudny, Russia; ^3^Insilico Medicine, Emerging Technology Centers, Johns Hopkins UniversityBaltimore, MD, USA

**Keywords:** neural networks, bump attractor, Hopfield networks, innate connections, self-organizing mapping, cortical column, dynamic attractor

## Abstract

In this work we reveal and explore a new class of attractor neural networks, based on inborn connections provided by model molecular markers, the molecular marker based attractor neural networks (MMBANN). Each set of markers has a metric, which is used to make connections between neurons containing the markers. We have explored conditions for the existence of attractor states, critical relations between their parameters and the spectrum of single neuron models, which can implement the MMBANN. Besides, we describe functional models (perceptron and SOM), which obtain significant advantages over the traditional implementation of these models, while using MMBANN. In particular, a perceptron, based on MMBANN, gets specificity gain in orders of error probabilities values, MMBANN SOM obtains real neurophysiological meaning, the number of possible grandma cells increases 1000-fold with MMBANN. MMBANN have sets of attractor states, which can serve as finite grids for representation of variables in computations. These grids may show dimensions of *d* = 0, 1, 2,…. We work with static and dynamic attractor neural networks of the dimensions *d* = 0 and 1. We also argue that the number of dimensions which can be represented by attractors of activities of neural networks with the number of elements *N* = 10^4^ does not exceed 8.

## Introduction

The idea of neural systems working as unification of many similar units (“hyper-columns”) exists in neuroscience for years (Horton and Adams, 2005). There are several approaches to understanding inner machinery of elementary neural networks. One approach involves revealing of neural connections experimentally. This is usually performed by identification of all connections in serial electron-microscopic slices of whole brain (Mikula et al., 2012). A recent molecular engineering approach by Zador et al. (2012) appears to simplify the problem. Other approaches try to match the observations with theory. One set of ideas theorized that most neural connections are formed by associative memory processes (Pavlov, 1927; Hebb, 1949; Marr, 1969, 1971). The most prominent is the notion of Marr's collateral network (Marr, 1971), which has been later re-discovered as the Hopfield network for associative memory (Hopfield, 1982). Network dynamics of the latter (although only in the case of symmetrical connections, which seems to be hardly possible in live neural systems) could be described by a potential energy function, which is minimized with the activity dynamics. Attractor states of Hopfield networks are isolated from each other. Continuous sets of attractor states present a completely different problem. We consider that two states *S*_1_ and *S*_2_ are connected, if ρ(*S*_1_, *S*_2_) ≤ 2 (ρ is Hamming distance). The connected set of stable states can constitute a grid of states, which can be used for representation in a brain of continuous variables. We will refer to the attractors, which can be used for the grids of the *d*-dimensional variables, as attractors with dimension *d*. So, the set of isolated attractor points (the set of stable states in the Hopfield network) has dimensionality *d* = 0. In (Dunin-Barkowski, 1984, 1986; Dunin-Barkowski and Osovets, 1995) the Hopfield-type neural networks, based on “continuous” sets of vectors, were considered. They can represent finite grids for one-dimensional variables (Dunina-Barkowska and Dunin-Barkowski, 1993). Hard-wired networks (networks with innate connections) with continuous attractors are known since (Amari, 1977). Recently, it has been discovered in isolated cortex slices experiments that many reverberating connections in cortex are innate (Harris and Mrsic, 2013). Also, the theoretical reasoning has been expressed that attractor neural networks can be innate and formed in ontogenesis with help of special molecular markers (Dunin-Barkowski, 2011a; Dunin-Barkowski et al., 2011). In this work we will present the advantages of using molecular marker based attractors for modeling basic types of neural information processing with computational experiments on attractors with dimensions *d* = 0 and 1. We will show the robustness of zero dimensional attractors to noise, we will show how can be visualized all states of preformed linear ring attractors (*d* = 1), and we will then analyze how the dimensionality affects the learning of an attractor network and present an extension of Kohonen's SOM. All the learning experiments will be done with McCulloch–Pitts Neurons. Some of the experiments with the activity dynamics in neural networks with innate connections will be extended to Leaky Integrate and Fire Neurons to show that the presented concept is not limited to one neuron model.

## Attractor neural networks

There are at least three general mechanisms for making attractor neural networks. The first is the self-obvious method (Amari, 1977). Here, the neurons are considered to be located in physical space and the connections are established in direct relation to the distance between neurons.

The second mechanism uses Hebb modifiable synapses. For *d* = 0, it was proposed in Marr (1971) and Hopfield (1982). For *d* = 1, it was studied in Amari (1977). To make the neurons of the network properly interconnected, they should be exposed to the signals from the external world for a certain period of time. This is provided by extensive scanning of the environment by the animal hosting the neural network (Hopfield, 2010; Samsonovich, 2014). In this “conditioning” process, the neurons, which get similar information from the external world, are often excited simultaneously. Due to Hebb-type learning rules, they become connected. Thus, in associative neural networks, the firing of each neuron is connected to the specific input information by the inborn connections.

Here, we propose and explore the third mechanism. The idea has been discussed earlier in Dunin-Barkowski (2011b). Our approach makes use of the following considerations. There is undisputed data showing that many inter-neuronal connections are inborn (Perin et al., 2011; Harris and Mrsic, 2013). The inborn attractor networks are obtained with help of connection rules, enabling neural networks to have attractors with desired properties.

In contrast to the attractor networks, based on Hebb synapses, the attractor neural networks with inborn connections inside them must tune their external connections to endow the neuron firing with a certain sense. We consider concrete examples of such processes later in the paper. In this paper, we deal with the networks with inborn connections inside the network and restrict analysis to the attractor dimensions *d* = 0 and 1.

### Molecular marker-based attractors, *d* = 0

Molecular markers can be used to get the matrix for the neural network with *M* isolated (*d* = 0) attractor points. There are *M*×*L* markers belonging to *M* classes with *L* elements in each class. The distance between markers is 0 when markers belong to the same class, and non-zero, say 2*L*, when markers belong to different classes. The markers are distributed randomly between the neurons so that each neuron gets *q* = (*M*×*L*)∕*N* markers, which all belong to different classes. For simplicity, we consider only cases when *q* is an integer while generalization to non-integer values of *q* is not difficult. Then, the neurons *i* and *j* are connected with excitatory connections only if the neurons contain markers of the same class. Contrary to the method of the learned connections, this method does not specify which concrete states belong to the attractor in advance. The set of attractor states depends on results of random distribution of markers between neurons. This connection rule provides mutually excitatory connections between neurons that have the same type of markers. This means that neurons with same type of markers might persistently excite each other. The state of the network when these *L* neurons are excited and the rest neurons of the network are silent, presents an element of the set of the neural network attractor states. This statement holds for each of the *M* types of molecular markers. Therefore, the attractor for the neural network in which neural interconnections are made with molecular markers consists of *M* states, *S*_*m*_, ($m=\bar{1,M}$) of activity of the network of *N* neurons. This is true (with the probability close to 1), while *M* does not exceed certain value, which depends on *N* and *L*. When *L* is small compared to *N*, the distance between any two states of the attractor is close to the value 2*L*. The interconnection matrix consists of 1 and 0. It is symmetric and has all zeros at the diagonal. The Hebb–Hopfield method (Dunin-Barkowski and Osovets, 1995) and the method of molecular markers result in the similar matrices. The only difference is in our knowledge of attractor states. In the first case, we initially select states of network activity which are to be the attractor states. In the second case, we randomly distribute markers between neurons while not knowing which neurons will be active in that or any another attractor state of the network.

More details on MMBAN with *d* = 0 are given in Supplementary Materials 2 and 5.

### Bump attractors *d* = 1

The first neural network with a continuous one-dimensional attractor has been was discovered by Amari (1977). He considered a set of neurons located along a line with excitatory local and inhibitory more distant connections (“Mexican hat”; Amari, 1977). In this network, there are stable states of activity in which neurons of a local group are active and the rest neurons are inhibited. This type of attractor is known as a “bump” attractor, as active vs. inactive neurons in the attractor state present a “bump” on a line of neurons. These types of attractors exist in neural networks of many types of neural models. For convenience, we give the general definition of this phenomenon below.

#### Definition of bump attractor

**Figure 1:**
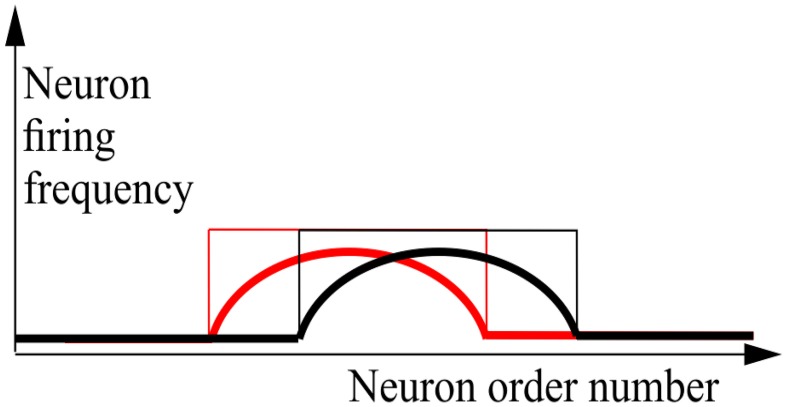
**Schematic drawing of activity plots for the neural networks with bump attractors**. Abscissa—neuron order number, based on the properly chosen enumeration of the neurons, ordinate—neuron firing frequency for continuous-valued neuron model (thick line) and the activity state (excited/silent—thin line) for MCP neuron model. Red and black lines refer to the neighboring stable states of the network. It is also supposed that many intermediate stable states (between the red and black states) of the same “waveform” as the pictured states, fill the interval between them. The figure visualizes the definition of bump attractors.

Consider a network of *N* MCP (Supplementary Material 1) neurons which are connected to each other. For handling the neural network, it is substantial that each of the neurons has an individual identity. Without loss of generality we can consider that these identities are order numbers, from 1 through *N*, each of which is permanently attached to a concrete neuron. This attachment provides neurons with their “individual names.” Ordering the neurons according to these numbers yields the basic order of neurons. Sometimes, it is convenient to use altered order numbers of the same neurons. A particular alternate enumeration of neurons can be presented as the set {*a*(1), …, *a*(*N*)}, which is a permutation of {1, …, *N*}. The *a*(*i*) can be also considered as a vector-function, i.e., a mapping of the set {1, …, *N*} into itself. Obviously, for *N* neurons there could be exactly *N*! enumerations. The state of the network, in which *L* neurons are permanently active and (*N*−*L*) neurons are permanently silent we name an attractor state of the network. Note that for any attractor state *s* = (*s*(1), …, *s*(*N*));*s*(*i*)∈{0, 1}, with *L* excited elements (*s* = 1) of any network, there exist such an enumeration {*a*(1), …, *a*(*N*)} that *s*(*a*^−1^(*L* + 1)) = … = *s*(*a*^−1^(*L* + *L*)) = 1, where *a*^−1^(*i*) is the reverse function to *a*(*i*). In other words, for any attractor state of the neural network, there exist such an enumeration of neurons with which all excited neurons are numerated sequentially, starting with order number (*L* + 1). This fact is indeed trivial, but we need it for further formulation. Now, we say that all attractor states of the network constitute the bump attractor, if for each attractor states of the network there exists such enumeration, *a*_*s*_, that for the states *s*_*i*_; (*i* = 0, …, 2*L*) keeps correct that *s*_*i*_(*a*_*s*_(1 + *i*)) = *s*_*i*_(*a*_*s*_(2 + *i*)) = … = *s*_*i*_(*a*_*s*_(*L* + *i*)) = 1 and these (2*L* + 1)states are attractor states of the network. This rather complicated definition can be clarified by the graphics of Figure 1. The case of continuously-valued LIF (Leaky Integrate and Fire) neurons is also shown here.

In Figure 1, states of individual neurons are plotted vs. their order number. For the neural network with bump attractors, for each attractor state there exists an enumeration with which there are at least 2*b* + 1 attractor states *b*~*L*∕2, each of which starts with excited neuron at order number *i* = *L*−*q* + 1 and continues with excited neurons through the order number *i* = 2*L*−*q*, (*q* = 1, …, *b*). Thus, each of these 2*b* + 1 attractor states in this enumeration looks like a “bump” of *L* successive active neurons, while the rest of neurons are silent. This definition is practically trivial for the network, where all neurons constitute a ring of the length *N* with excitatory connections between adjacent neurons. In these rings, the number of attractor states (the “length” of the ring of attractor states) coincides with the number of neurons in the network *N*. Later in this paper we will demonstrate that “the rings” of attractor states can be of the length *kN*, where 1 < *k* < *K*(*N*), with *k*(*N*) linear growing with *N* with constant *L*. In other words, the fact that a neural network has a bump attractor means that the set of its attractor states can be presented as locally linear in vicinity of each of its attractor states. To observe this presentation, the appropriate enumeration of neurons should be selected. In general case, the necessary enumeration depends on the concrete state in vicinity of which we make observable the local linear structure of the attractor. However, for some types of bump attractors, there do exist enumerations which yield linear representation for substantial part (1∕*R*)-th of all attractor states; *R* = 1, 2, 3, … depending on the case; (Dunin-Barkowski, 1986; Dunin-Barkowski and Osovets, 1995). In this paper, we do not consider bump attractors with dimensions *d* > 1. However, the generalization of the bump attractor definitions to these cases can be more or less straightforward.

The bumps can be either stationary for stationary attractor states (Dunin-Barkowski and Osovets, 1995), or propagating over the line of neurons in case of dynamic attractors (Dunin-Barkowski, 1986; Dunin-Barkowski and Osovets, 1995). The formal definition for dynamic bump attractors can be simply obtained from definition of a static bump attractor. In the case of a dynamic attractor, the velocity of propagation of the bump over the line of neurons can vary, depending on the excitatory or inhibitory background (Dunin-Barkowski, 1984; Dunin-Barkowski and Osovets, 1995; Grossberg and Pilly, 2014). The number of attractor states in bump attractors can exceed the number of neurons (Dunin-Barkowski, 1984). The properties of learned bump attractors, which emerge in process of network connection forming after learning in the network of activity patterns, are described elsewhere (Dunin-Barkowski, 1984; Dunin-Barkowski and Osovets, 1995). Next section deals with inborn bump attractors.

#### Pre-formed attractors, *d* = 1

An inborn mechanism to obtain neural networks with *d* = 1 bump attractors has been proposed (Dunin-Barkowski, 2011b; Dunin-Barkowski et al., 2011). Computational tests of these mechanisms have been first tried in Solovyeva (2014). This network consists of *N* interconnected binary neurons. Its state is characterized by *N*-dimensional vector of “0” and “1.” The interconnection matrix **T** (*N*×*N***)** is formed with the help of model molecular markers, as is described below. For network dynamics computing we use the asynchronous random dynamics, which is defined in Supplementary Material 1. The general idea of molecular markers in this section and in Section Molecular Marker-Based Attractors, *d* = 0 is similar, but the details differ, and help in building a neural network with attractors with *d* = 1, instead of *d* = 0.

**Figure 2:**
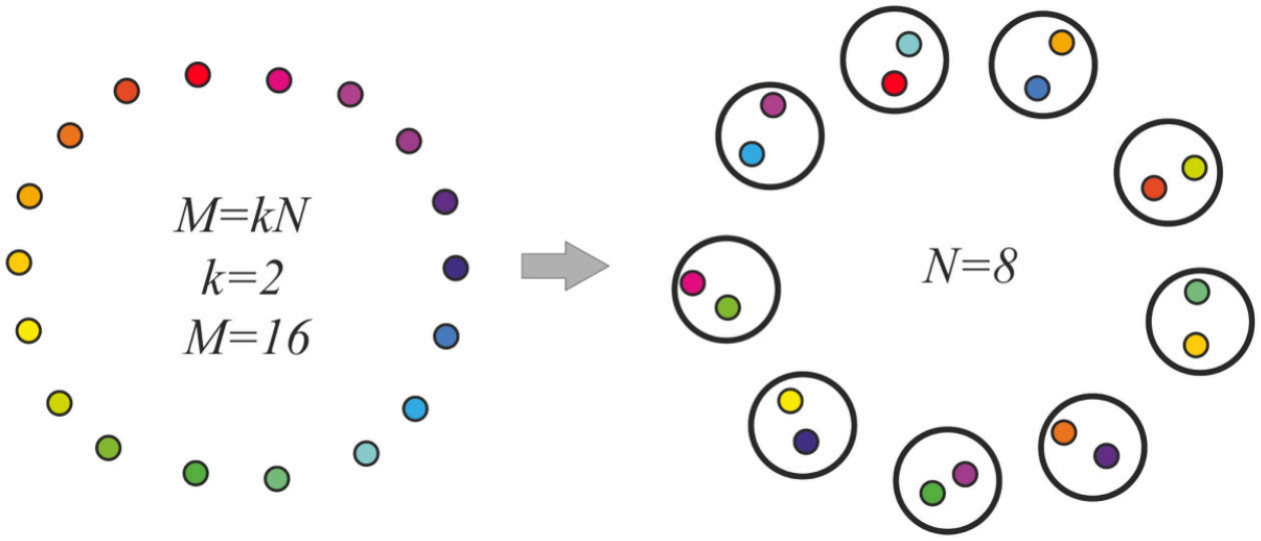
**Model molecular markers (left) and their random distribution between neurons (right)**. The molecular markers are shown as small colored circles. Distances between the markers are indicated as color resemblance. Larger circles are the neurons, each of which gets two molecular markers of substantially different colors. The inter-neuronal excitatory connections are made between the neurons, which have markers of similar colors.

Here, the model molecular markers μ_*h*_; *h* = 1, …, *M* are considered to make a ring, so that the marker next to the marker μ_*M*_ is μ_1_. For the distance between markers μ_*i*_ and μ_*j*_, we take *D*_*ij*_ = min{|*i* − *j*|, (*N* − |*i* − *j*|)}. We assume *M* = *kN*, with integer *k*. Then *M* markers are distributed between *N* neurons randomly, providing each neuron an even number of markers, *k*. Besides, for markers, which fall into one neuron, we demand that the distance between the markers exceeds a fixed value, Δ. This process is schematized in Figure 2. Here, the resemblance between the markers is denoted by the colors of the “molecular markers.”

The distribution of markers in neurons is performed by placing one marker at a time into one neuron with the help of random number generation and necessary checks. This process either yields a valid distribution of markers in a limited time, or it does not. The larger the values of *N* and the smaller *M* and Δ are, the sooner the distribution process is completed. After completion, those neurons, which have markers with distance < δ, establish excitatory connections with each other with connection weight value of +1. The rest neurons are also connected with inhibitory connections of the weight –σ. The neurons are not connected to themselves. In the network, which has been formed as described, there are stationary attractor states. Several methods can be used to characterize the structure of the set of attractor states. The first one uses statistics of the states, into which the network gets after transients (“relaxes”) from randomly chosen states. It is well known that the network with symmetric connections gets to attractor states in ~lg(*N*) time steps (Hopfield, 1982). The second method makes use of “artificial” dynamics introduced into the neural network. In this case, the neural threshold is made dependent on the integral value of the recent activity of the neurons (Dunin-Barkowski and Osovets, 1995). Due to the threshold accommodation, the activity of neurons tends to shift from a current to an adjacent state. This method enables the activity of the network to “slide” over any connected chain of states which exists in the neural network. In case of the closed (“circular”) chains, the activity can circulate over the circles for an indefinitely long time. It should be stated here that in this paper we are dealing with sets of markers and sets of states, which compose rings. However, the ring structure is used here only for convenience of avoiding setting of boundary conditions in the beginning and the end of the chains of states or markers.

#### Extension to kohonen's SOM

As has been stated in the beginning of Section Attractor Neural Networks, the neural networks, which have inborn inter-neuronal connections, must tune their external connections to endow the neuron firing with certain sense. Our approach is based on a natural extension of Kohonen's SOM approach (Kohonen, 1982) to neural networks, which have one-dimensional attractor state.

The model consists of *R*-dimensional input space and a neural network of *N* neurons. For convenience, we consider that *R* input “receptors” read out the cyclic input variable, φ, which might be (for example) the animal's head direction angle 0 ≤ ϕ ≤ 1; as φ is cyclic, ϕ = 1 represents the same direction as ϕ = 0. At a given value of angle φ, receptors, which are broadly tuned to this angle, are excited. The tuning curve of each receptor is bell-shaped with a definite width. The time is discrete. Values of the input variable in successive moments of time are independent and randomly selected.

In the naive system, all receptors are connected to all neurons of the targeted neural network, and connection weights are randomly chosen. The neural network consists of *N* binary neurons with recurrent symmetric connections. In computational experiments, two types of recurrent networks are used. In the first series, we use the network described in Section Pre-formed Attractors, *d* = 1 with *k* = 1. We will refer to such type of networks as a networks with a full ring attractor. In the second series, we use the network which has two independent full ring attractors (Dunina-Barkowska and Dunin-Barkowski, 1993; Romani and Tsodyks, 2010). Such networks have two independent (random in relation to each other) ring attractors of length *N* each.

The learning process includes the following steps:

1. The value of the input variable is selected.
2. The receptors are excited according to their tuning curve, yielding the *R*-dimensional input vector for the neural network (no activity in the network).
3. The neurons are activated due to input signals from the receptors via the connection matrix.
4. In the series of iterations, the states of the neurons of the network are updated, starting with the state, obtained at the previous stage, in accordance to the neural network equations for 20 units of time (no working receptor connections, network activity turns to stable in 3–6 time steps).
5. The connections from receptors to the neurons are modified and stages 1–5 are repeated.

The rule for modification of connections is following:

(1) $$W_{i:}=W_{i:}+\eta \cdot ({\left(XV^{T}\right)}_{i:}-W_{i:})$$

This expression means that *i*-th line of the *R* × *N* matrix **W** is slightly turned (as scaled with a small parameter η) in direction of the input vector, **X**, which has elicited the current state of the network activity, **V**.

The modification of connections continues until it could be obvious that the matrix **W** yields continuous mapping of the variable, which is sampled by the receptors into the attractor states of the neural network. This conclusion is made based on a visual observation of the matrix **W**.

## Numerical experiments and results

### Robustness to noise, *d* = 0

**Figure 3:**
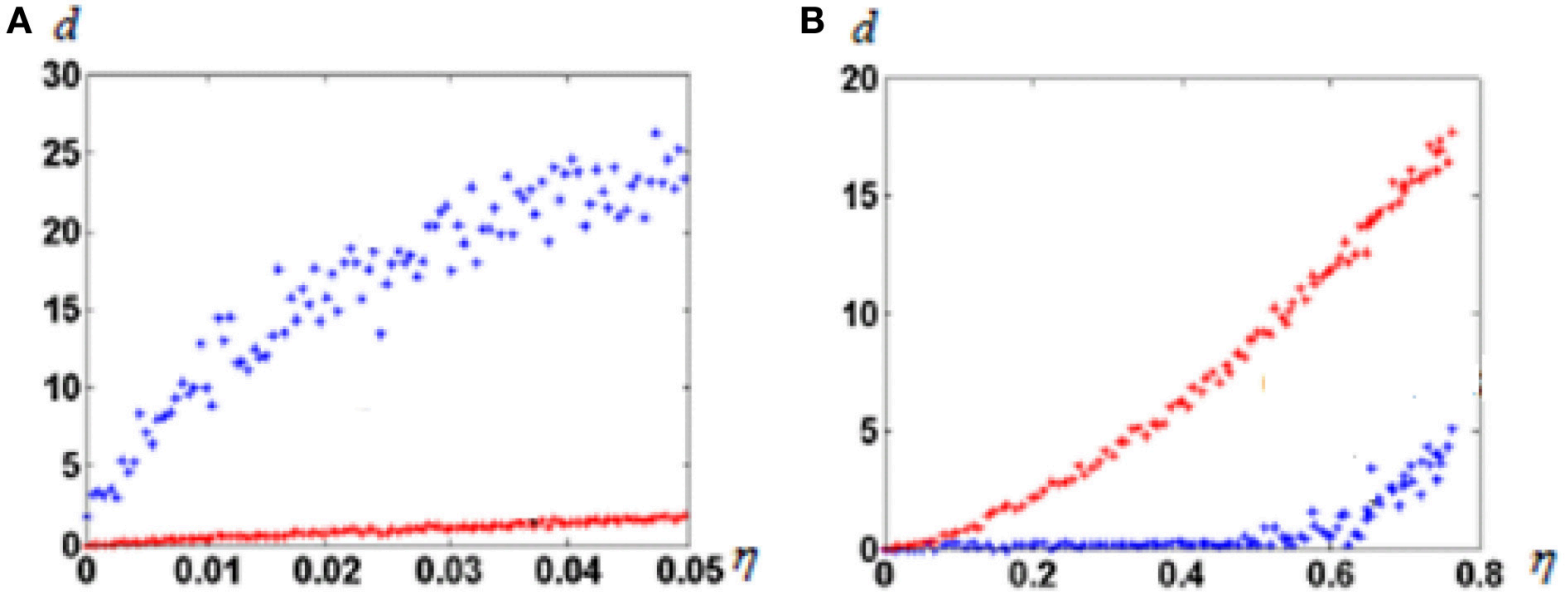
**Noise dependence of “output error” of neural network responses**. **(A)** Before learning; **(B)** After learning. Blue dots, attractor neural network; red dots, not connected neurons. Note the huge difference of abscissa scales between **(A)** and **(B)**. *R* = 100; *N* = 300; *M* = 100; *L* = 20. Note that before learning the attractor neural network enhances the input error. That happens because noise might force neural network to get to other attractors than imposed by a noiseless signal. The Figure demonstrates that the learned attractor network has strong noise tolerance.

In this experiment we observe effects of noise when input vectors are randomly chosen. First, we construct the attractor neural network with *M* “isolated” attractor points, as described in Section Molecular Marker-Based Attractors, *d* = 0. Then, we select and fix *M* random vectors in *R*-dimensional space. All coordinates of vectors are selected randomly from the interval [–1, 1]. Initial values of connections from input fibers to the representing neural network are random. One by one, we feed all selected random vectors to the input fibers and memorize the states of the neural network to which network states converge after starting from input from each selected input vector. The set of the selected input vectors is further checked as follows. If the newly fed preselected vector imposes convergence of the neural network into the state, which has been already memorized for a previously fed vector, we select a new random vector with which the system converges to the previously non-“occupied” attractor state. When the selection procedure is complete, each of the attractor state has a corresponding to it random vector in the input space. Afterwards, we feed the same input vectors with noise added to the network. Figure 3A shows results of tests of the network with the selected *M* vectors and with same vectors with added noise. In this case, to each selected vector *V*_*i*_ we added noise, i.e., the *R*-dimensional random vector ξ_*i*_, which coordinates are random in the interval [−η, η], where η∈[0, 1] is the noise amplitude. Two types of the network are compared. One of them is the network with *M* (“isolated”) attractor points. The other is the network of non-connected neurons. From those “non-connected” neurons for the given input vector, *L* neurons, which receive maximum excitation, are set excited.

For learning of connected and not-connected networks, the modified Rosenblatt's perceptron rule was applied. Perceptron is a device, which for each of the selected vectors *X*_*t*_ calculates values *O*_*t*_:

(2) $$O_{t}=\sum_{\;i=1}^{\;R}w_{\;i}X_{\;ti}$$

where *w*_*i*_ are the tunable real-number-valued parameters of the perceptron. The teacher compares the sign of *O*_*t*_ with *y*_*t*_. If they coincide, the vector of *w*_*i*_ remains the same. If they differ, the vector of *w*_*i*_ obtains the new value (Rosenblatt's rule):

(3) $$w=w+X_{t}y_{\;t}$$

The perceptron learning is fast. It is known that if this iterative learning process converges to fixed values of *w*_*i*_ the final state is attained after only a few iteration steps (Rosenblatt, 1958). We use a modified perceptron learning rule as described below. In each cycle of work of the system there are two phases of functioning. In the first phase, the input vector *X*_*t*_ acts on all neurons of the network, imposing a state of excitation on some of them. *L* neurons, which get maximum excitation from the input, are left excited at this phase. The second phase of the work of the system is a relaxation of the system from its initial state, to one of the attractor states of the network. Afterwards, the connection weights of the neurons with the external fibers are modified. For those neurons in which the initial states coincide with the final states, no actions are undertaken. For neurons, which initial state was 0 and final state 1, the *X*_*t*_ vector is added to its external connections vector. For neurons, which initial state was 1 and final state is 0, the *X*_*t*_ vector is subtracted from its external connections vector. This procedure is repeated for all input vectors *X*_*t*_ until matrix *W* keeps changing. In our computations, the number of iterations in all cases was <50.

As can be seen from Figure 3B, learning dramatically changed the noise dependence of the attractor neural network. After learning, up to very large noise, neurons keep discharging with the same pattern. The behavior of uncoupled neural network does not substantially depend on learning (note huge scale difference in abscissa of Figures 3A,B).

We explored this phenomenon in a wide range of parameters. In all cases, qualitatively, the behavior was the same. Neural networks with attractors demonstrate high tolerance to noise (up to 50%), while uncoupled networks are not resistant to noise. The revealed phenomenon, although transparent in its mechanisms, clearly demonstrates salient advantages of attractor neural networks with *d* = 0.

### Preformed attractors, *d* = 1, visualization of ring attractors

**Figure 4:**
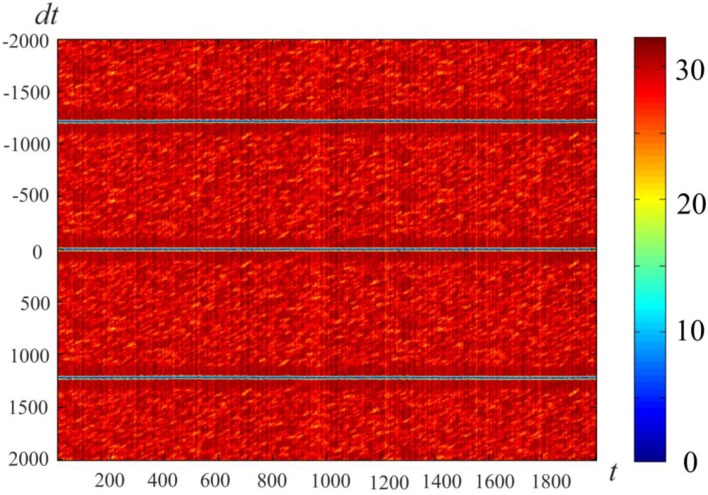
**Activity visualization (L-plot, see text for detail) in the network with connections, based on the ring of markers**. *N* = 300, *M* = 900, Δ = 80, δ = 12, σ = 3, mean value of *L* over the observation period, $\bar{L}=15$. *t* and *dt* represent time (in time steps of modeling); Δθ = 0.1, τ= 200, θ_0_ = 0. Color of Figure's points (color code is shown at the right strip) indicates the distance between state vectors of the neural network in different time moments, i.e., between the (*N*-dimensional) state vectors *V*(*t*) and *V*(*t* + *dt*). The Figure shows that the states of the network are repeated with the interval *T* = 1225.

Figure 4 gives an example of activity in a neural network of 300 neurons, whose connections were made with a help of a ring of 900 markers as described in Section Pre-formed Attractors, *d* = 1. We introduced “artificial” dynamics in the network, as described in Pre-formed Attractors, *d* = 1. The plot color codes the distance (in configurational space) between the current state of the network and the states in the past (above the mid-line) and the future (below the mid-line). This method of visualization of multidimensional processes, *L*-plot, was described in Dunin-Barkowski et al. (2010).

**Figure 5:**
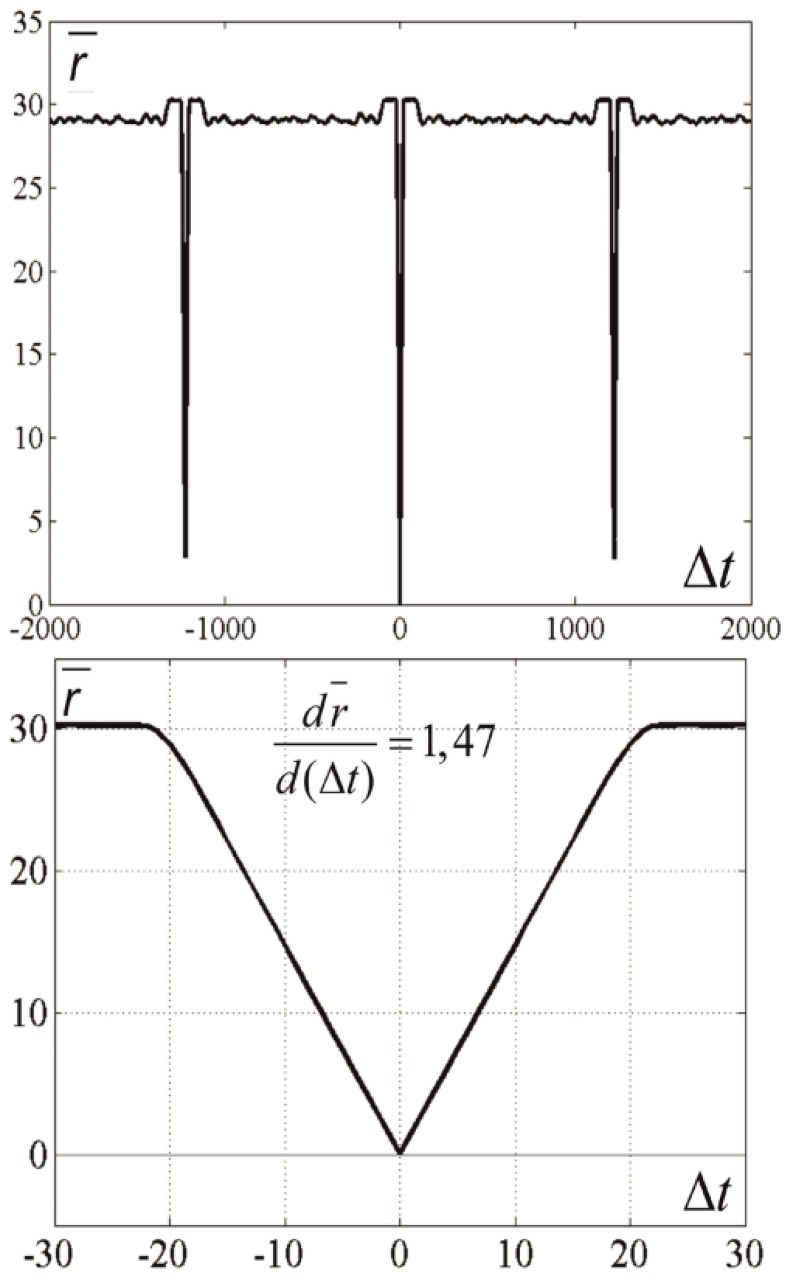
**Average distance between the current state and past and future states**. Top and bottom differ in time scale. *N* = 300, *M* = 900, *T*_*net*_ = 1225. The Figure is obtained by averaging the data, plotted at Figure 4 over the horizontal axis. Ordinate—distance between the states of neural network *V*(*t*) and *V*(*t* + Δ*t*), averaged over *t*-values of Figure 4. It is first obvious that the “periodicity” of activity in the network is noisy: the distance minimums at *T* = ±1225 are not zeros. It is also obvious that the dependence of distance on time difference is linear, when distance is close to zero. This fact makes well-defined the velocity of propagation at |Δ*t*| < 15. This fact enables “counting the number of states,” which activity of the network attends over the cycle of periodic activity, in this case *M*' = 900.37 ≈ 900 = *M*.

The horizontal lines above and below the mid-line show that the activity in the network is cyclic, with the period of *T*_net_ = 1225 time steps. The periodicity of the neural network activity means that there is a closed chain of attractor states in the network, all of which are attended by the system in cyclic dynamics. The period of the cyclic activity is determined by two factors: (a) the number of states attended by the system, and (b) the rate of threshold accommodation. The plots of Figure 5 are obtained by averaging of the plot of Figure 4 over the horizontal axis. Top and bottom graphics show the same data with different time scales. At the top plot the main feature is presence of the central “negative impulse” and its two symmetric replicas “in past and future.” They simply reflect the (almost) periodic processes in the neural network. At the bottom of Figure 5, one can see that in the time segment −10 ≤ Δ*t* ≤ 10 the derivative, *dr*∕*dt* is in fact constant, changing sign at *t* = 0. The important fact is that the product of *T*_net_ · (*dr*∕*dt*) in this case is 1225·1.47 = 1800.75, which practically coincides with the value 2*M* = 2·3*N*, the doubled number of markers, which were used for forming connections in the network in this case. In other words, this neural network has *M* attractor states that are connected into the ring chain of states with a minimal distance *D*_min_ = 2 between adjacent states. All of them can be visited sequentially if neurons have the property of threshold accommodation. The form of the curves at Figure 5 can be qualitatively explained. First, we explain the initial linear growth of the distance from the given state to the subsequent attractor states as a function of time. The growth is due to the fact that all attractor states have the same value of *L* (the number of “ones” in state vector), and the activity sequentially runs over all attractor states. The plot in fact means that activity propagates in the ring of attractor states with the same local properties, as activity propagates over a line of neurons.

As this linear increase of *r* continues almost until *r* = 2*L*, a slight distortion of linearity can be seen close to the value *r* = 2*L*. The distortion is due to the noisy influence of the remote parts of the ring of attractor states onto the interstate distance. Afterwards, the distance remains equal to 2*L* (which would be exactly the same as in case of activity propagation in a linear chain of neurons). However, the distance drops to another level, *D*, when the number of the states, passed by the network activity, from the reference state approaches Δ. In computational experiment, we have *D* = 29.06. Theoretical estimates yield the following expression for *D* (Supplementary Material 3):

(4) $$D\approx 2L\left(1-\frac{(r-1)L}{M}\right)$$

for *L* = 15, *k* = 3, *M* = 90 (Equation 4) gives *D* ≈ 29.06, in good accordance with the experiment.

**Figure 6:**
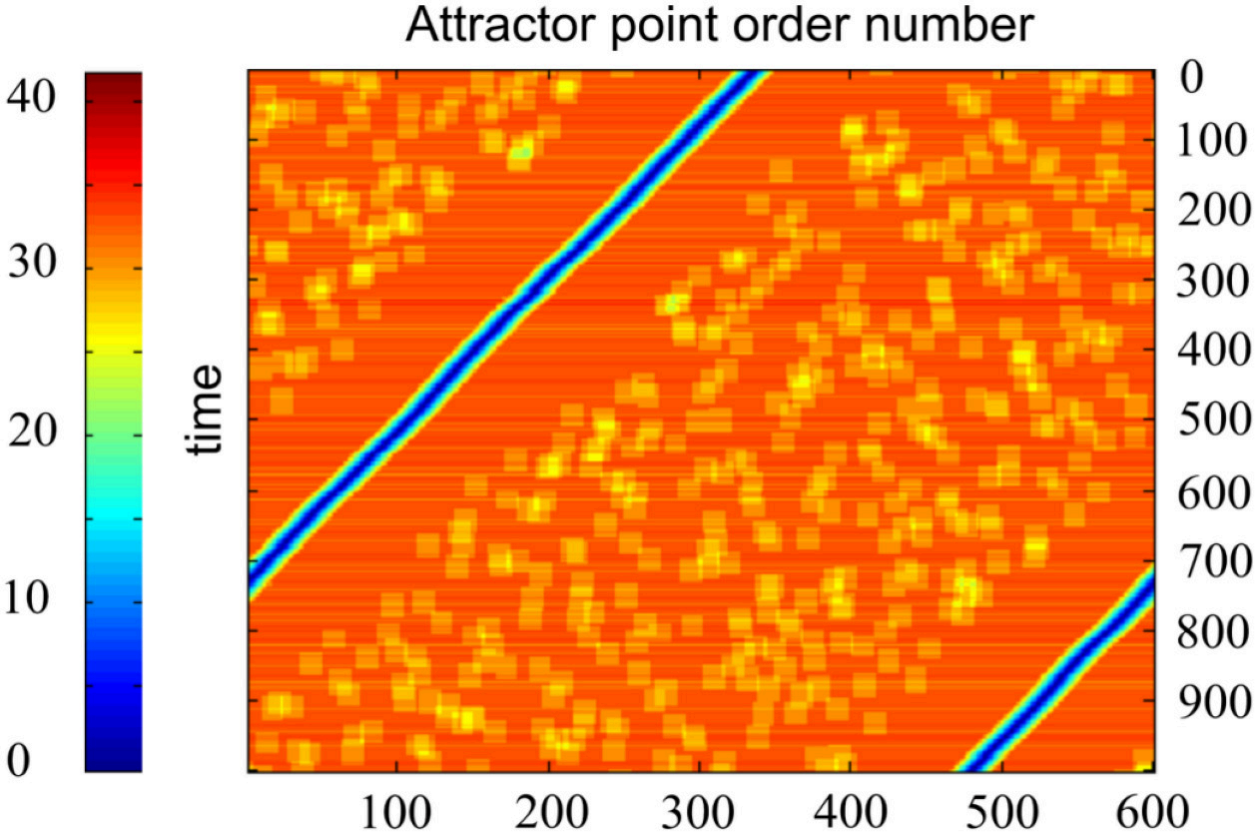
**Time plot of distances between the current activity state and candidate activity states, enumerated by the marker order number**. One horizontal line corresponds to one time moment. Abscissa—network state order number; ordinate—time. Color code for each point of each line shows how close is activity of the network to the state, which order number is at the abscissa. *N* = 300, *M* = 600, *D* = 80, δ = 12, σ = 3, other parameters are the same, as in Figure 4. The Figure demonstrates that the activity of the network in fact attends the candidate states, enumerated at the abscissa.

Another way of activity visualization in this type of neural networks is presented in Figure 6. Here, abscissa gives the order number of the specially selected M neuron states. Each of them includes *L* excited neurons, which contain markers with order numbers *i, i* + 1, *i* + 2, …, *i* + *L*−1, with *i* = 1, …, *M*. Ordinates give discrete time, which increases from top to bottom. The color of the point (*i, t*) at this plot gives the distance between the current state *s*(*t*) of the neural network and the state *s*(*i*) as defined above. Figure 6 shows that, in fact, the network states “slide” over the set of the selected states. The plot of the type of Figure 6 is characteristic for the computational experiments with sufficiently small value of *k* = *M*∕*N*. For larger values of *k* the picture of activity propagation is different.

### Activity of neural networks for different values of *k*

**Figure 7:**
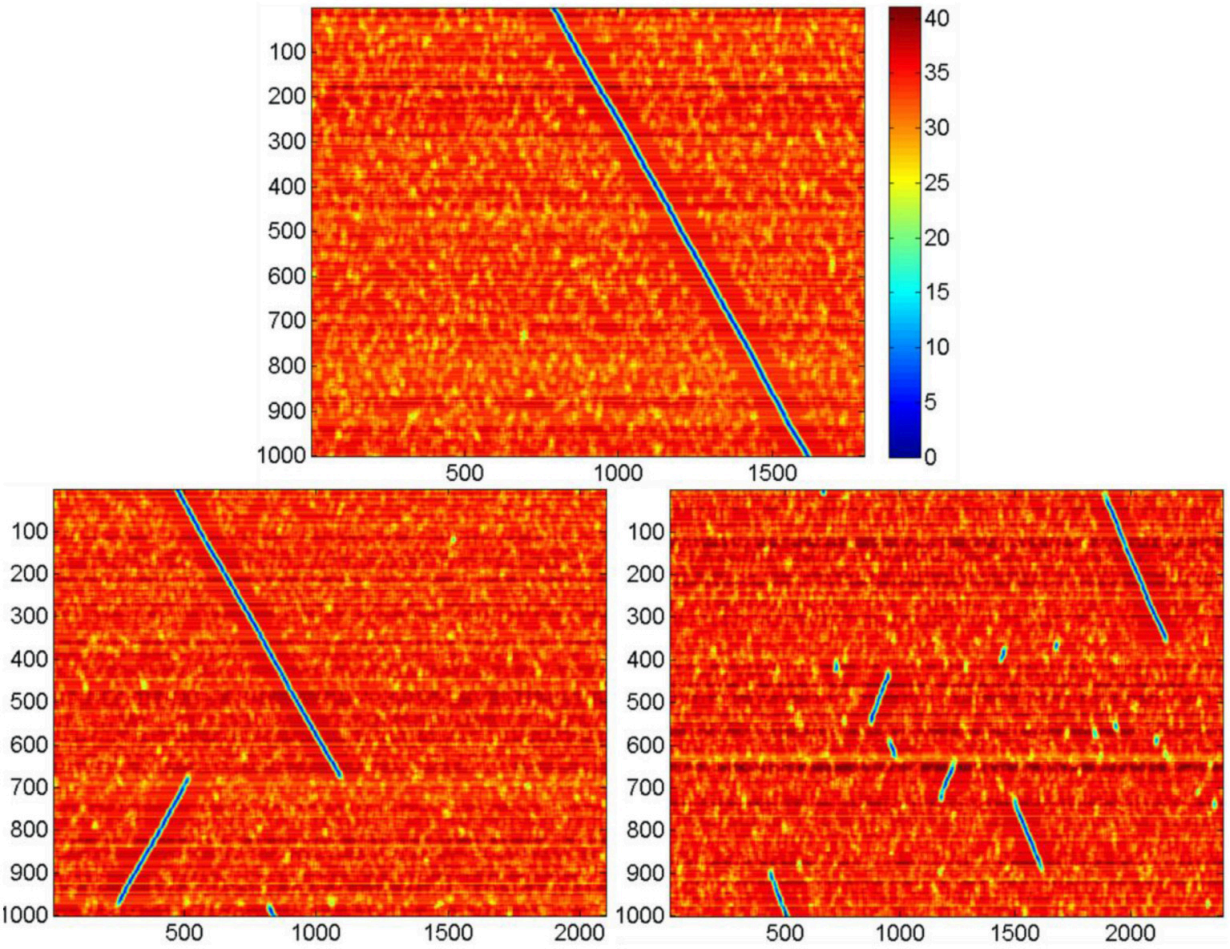
**Activity display for the neural networks formed with different values of *k***. Notations are the same as in Figure 6. *N* = 300, *D* = 80, δ = 12, σ = 3, Other parameters are the same, as in Figure 4. Top: *k* = 6, in this case the activity runs in cycles over all *k*·*N* attractor states (not shown); bottom left, *k* = 7; bottom right, *k* = 8. Note that for large *k* the activity jumps between points of attractor significant distances. Each leap of activity is accompanied with change of direction of wave propagation of the ring of attractor states (the incline of blue lines at the figures switches from negative to positive and vice versa after each leap. The number of jumps for the fixed time period increases with values of *k*. The figure illustrates the method of obtaining data for the Figure 8.

**Figure 8:**
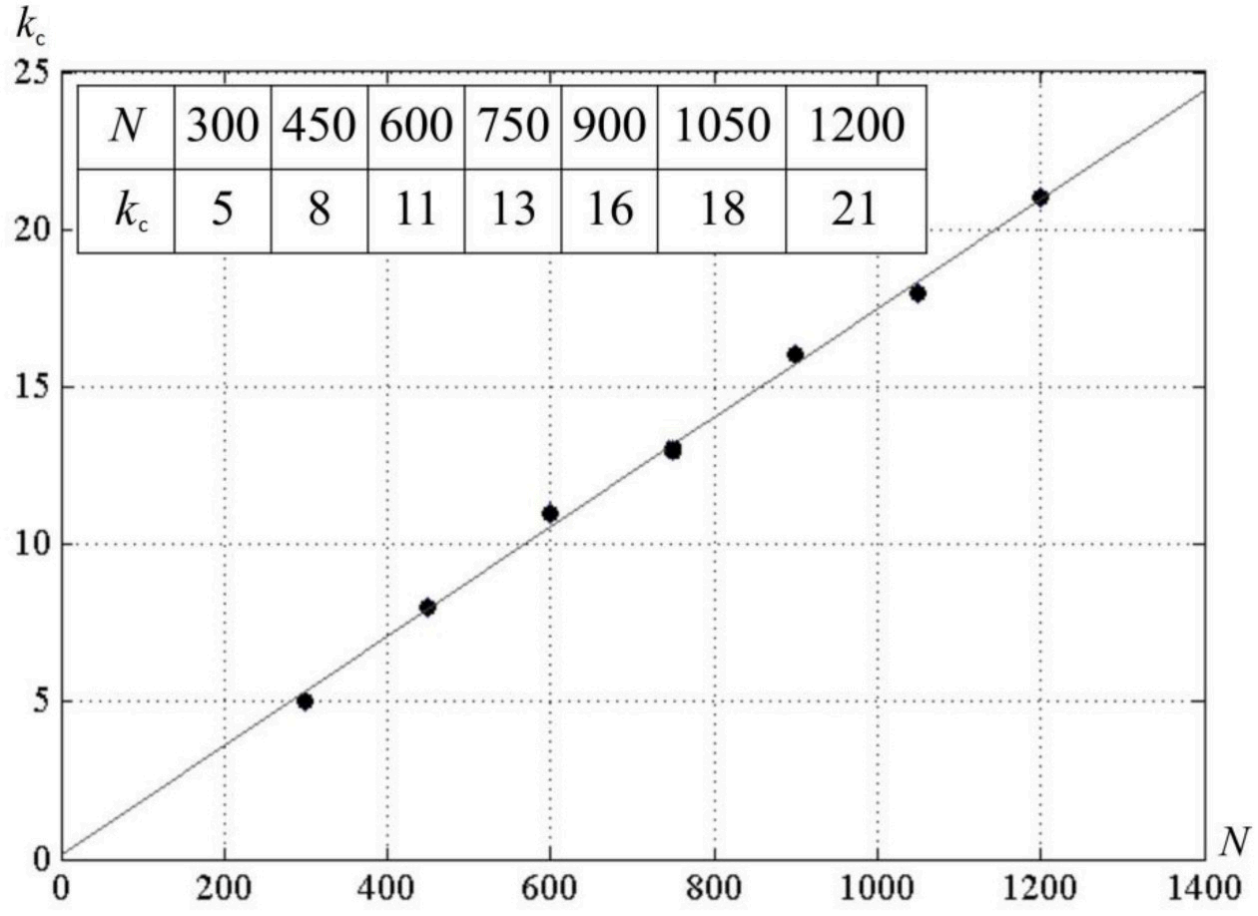
**The dependence of *k*_*c*_ on the number of neurons in the neural network *N***. *D* = 80, δ = 12, σ = 3; a line is a least square match of the computational experiment data.

We studied effect of *k* on network activity. With larger *k*, the neural network activity display can show the pattern given in Figure 7. It can be seen that for *N* = 300, there exists a critical value *k* = *k*_*c*_, such that for *M* < *k*_*c*_ · *N* the network activity follows the pattern displayed at Figure 6. For the larger *k*, the pattern changes. Screening of the parameter values in computational experiments yields the dependence of *k*_*c*_ on *N* (Figure 8). It is practically linear. An analysis gives the following expression (Supplementary Material 4):

(5) $$k_{c}(N,\Delta ,\delta ,L)=\frac{N}{2\delta \sqrt[{\;L}]{N}}$$

Correction factor$\sqrt[{L}]{N}$ in the denominator is between 1.0 and 2.0 for *L* ≥ 20 and *N* ≤ 10^5^. The correspondence between theory and computational experiment is fair.

### SOM type learning in static bump attractors

**Figure 9:**
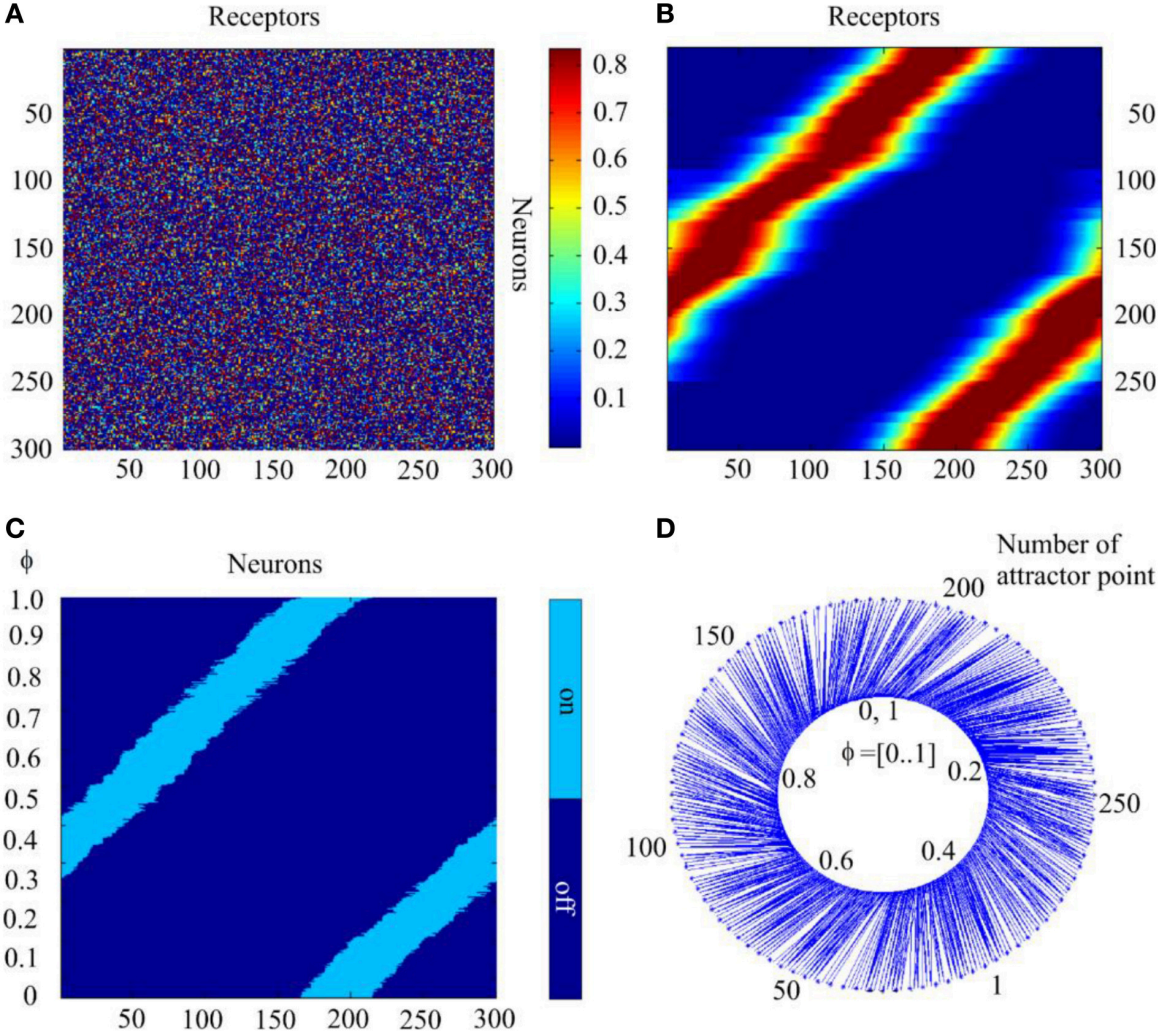
**The self-organization of mapping from receptors to the attractor network**. **(A)** Initial state of the matrix of the connections between the receptors and neurons of the attractor network. The value of matrix elements is color-coded. **(B)** The same matrix after completion of self-organization. **(C)** States of the network to which the activity of the network converges when the parameter of input signal takes 1000 sequential values in interval [0, 1.0]; abscissa—order # of neurons, ordinate—values of the parameter; light or dark blue colors denote excited or silent neurons. **(D)** Schematic mapping of the input signals to the states of the network. Inner circle, input signal parameter; outer circle, the attractor state order #; blue lines connect input signal representation points with the attractor points, to which neural network activity converges with the given input. The Figure demonstrates feasibility of SOM with neural attractors.

With the learning procedures described in Section Bump Attractors *d* = 1, we demonstrate here results of the computational experiments. Figures 9A,B show connection matrices before beginning and after completing the learning process. In the initial state, the matrix presents a random mosaic. It should be noted that the order numbers of receptors are given according to the variable values to which the receptors are tuned. Figure 9B shows the fact that learning **W** implements the continuous mapping of the sampled variable into attractor states of the neural network. Figures 9C,D show the results of testing the learned system. The test consisted of sequential presentation to the system of φ-values in the range [0, 1] with 0.001 steps. With each φ-value, the neural network relaxed for 20 steps of time (to ensure that all transients are over) to an attractor state. In Figure 9C the final state of the network for each φ-value is represented by a line with light blue points for active neurons of the final state and dark blue points for the silent neurons. It can be seen that the sequential test of a 1000 values of φ in the learned system yields activation of the sequential attractor states of the neural network. Figure 9D shows this result rather differently. Here, the inner circle of the figure represents the φ-values. The outer circle represents the order number of the neural network states, to which the activity of the neural network converges when it is activated with the concrete φ-value. The latter are connected to the former with thin lines. This form of the graphic display of the mapping of the input variable φ-onto the states of the neural network activity shows more detail than the method in Figure 9C. In particular, it can be seen that there are some deflections from the linear relations between the φ-values and the attractor states.

**Figure 10:**
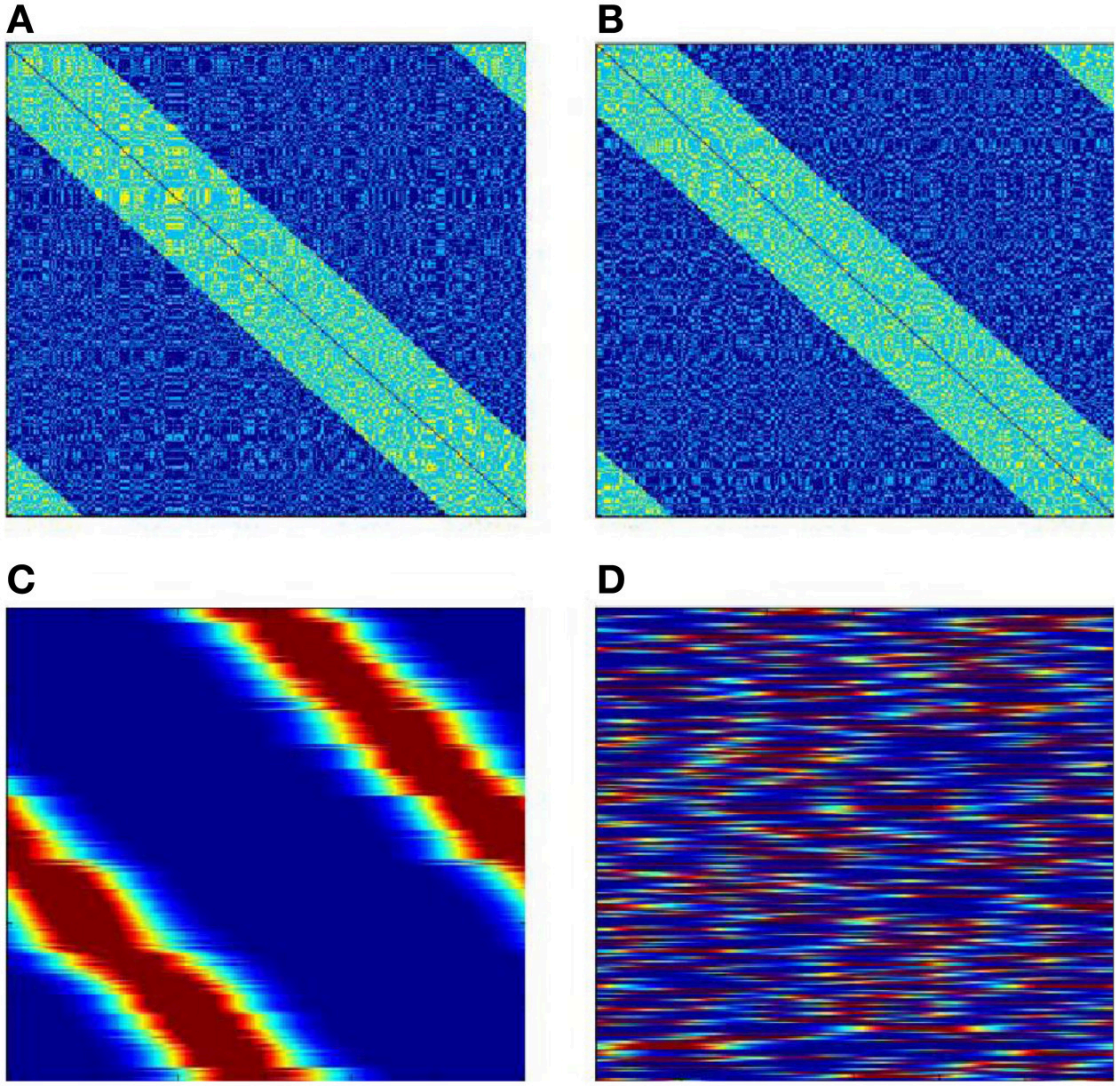
**SOM with two possible attractors for mapping input signals**. **(A,B)** are two views of neural network interconnection matrix **T** in the first in the second enumerations (the dimensions of the figures are *N* × *N*; the connection weight is color-coded: dark blue, −10; light blue, 1; yellow 2. **(C,D)** the connection matrix **W** after completion of learning, **(C)** neurons are numerated in the first enumeration; **(D)** neurons are numerated in the second enumeration. The number of neurons *N* = 300, the number of receptors *R* = 300. The Figure demonstrates that of two existing circular bump attractors, which exist in the neural network, the SOM process “selects” only one to use for mapping of the input signals.

Figure 10 shows results of learning for the case when the neural network has two independent full ring attractors. The inter-neuronal connections are formed with help of two sets of molecular markers. Each set has *N* elements with circular topology. Both sets are distributed randomly between the neurons so that each neuron gets one marker from each set. The excitatory connections are made between neurons which have markers (of either type) with distances less than δ. Figures 10A,B give two views of the neural network interconnection matrix. These views are obtained with two different enumerations of the neural network neurons. The two enumerations correspond to two different sets of markers. The views seem to be identical, but they are in fact completely different in their fine details. Figures 10C,D give two looks at the matrix **W** between the receptors and the neural network after learning. For the neurons, the enumerations of Figures 10A,B are used for Figures 10C,D. The first view shows that the learning has provided mapping of the variable φ onto one of the ring attractors of the neural network. Which of the two attractors finally “accepts mapping” depends on the random initial conditions.

#### Leaky integrate and fire model

In this section, we compare the computed behavior of the neural networks of McCulloch–Pitts neurons described in previous sections with the behavior of the networks of Leaky Integrate-and Fire (LIF) impulse neurons.

**Figure 11:**
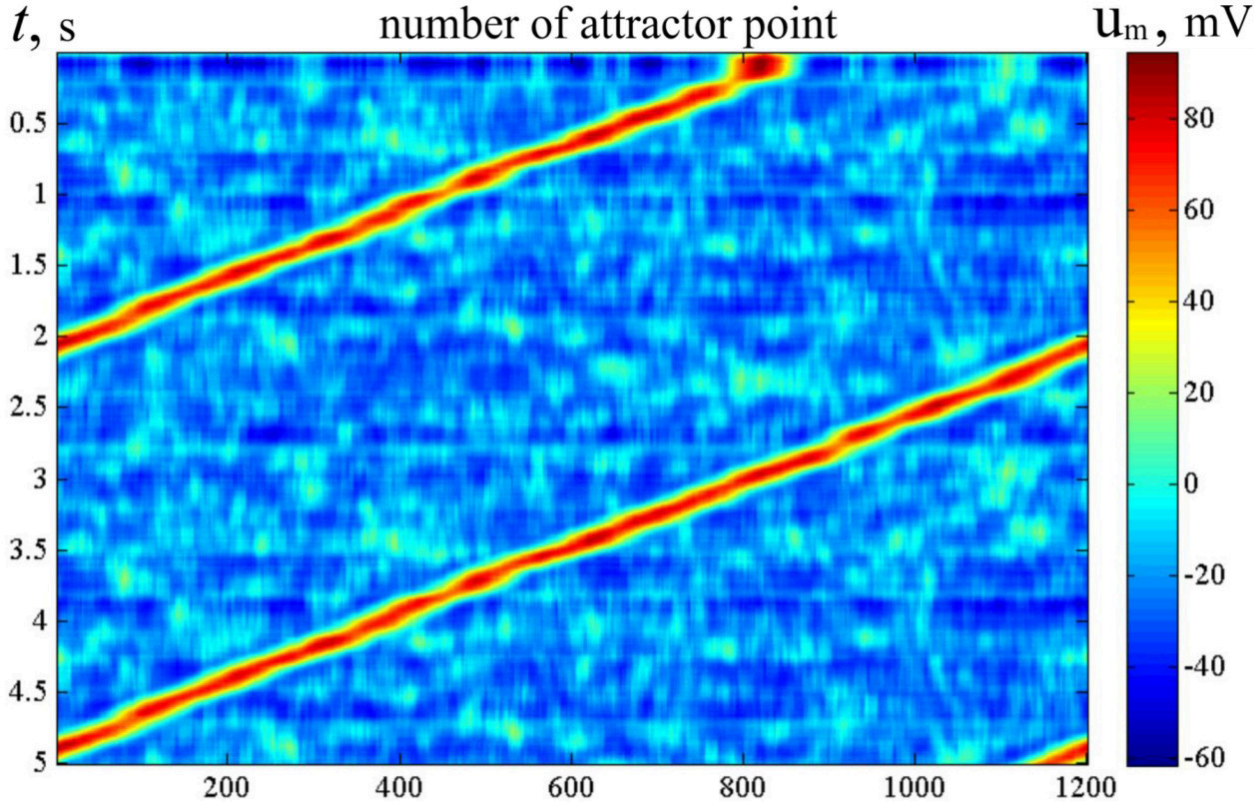
**Raster of activity for LIF bump attractor**. Horizontal line—order number of the molecular markers. Vertical—time (s), from top to bottom. The color of the dots codes the sum of membrane potentials of the neurons, which contains markers with order markers from [*x*−*L*∕2] to [*x* + *L*∕2], *x* = 1, …, *M*. LIF neural network with accommodation (Supplementary Material A1.3), τ_Ca_ = 0.1*c, N* = 600, *M* = 1200, *L* = 30. The figure looks like Figure 6. Alike the latter, it means, that the neural network activity of the neural network with one-dimensional attractor “rolls over” the candidate states in course of artificial dynamics (in this case, due to Ca^++^ entering into the neuron in connection with excitation impulses). The difference between Figure 4 and this figure is in the criterion of activity presence in a particular state (see the text for detail).

Figure 11 shows the activity dynamics in the network of LIF neurons. The excitatory neural network connections are made with the help of molecular markers, similar to the technique used with MCP neurons whose activity is shown in Figure 7. The inhibitory neurons get excitatory connections from all excitatory neurons, and they send their connections back to all excitatory neurons. In Figure 11, the color code of the pixels indicates the sum of the membrane potentials of the neurons in each of the *M* standard neuron sets (SNS) (def. below). The horizontal coordinate is the order number of the SNS, while the vertical coordinate indicates time. The standard neuron sets are those, which include the neurons with the markers (*j* − *L* ∕ 2), (*j* − *L* ∕ 2 + 1), …, *j*, (*j* + 1), …, (*j* + *L*∕2), for *j* = 1, …, *M*, where *L* = δ. The neurons have an accommodation property. Due to modeled calcium currents, the threshold of the neurons depends on their recent activity. Figure 11 clearly shows that the activity of the network slides with time over the ring of the SNS.

#### Dynamical neural attractors

**Figure 12:**
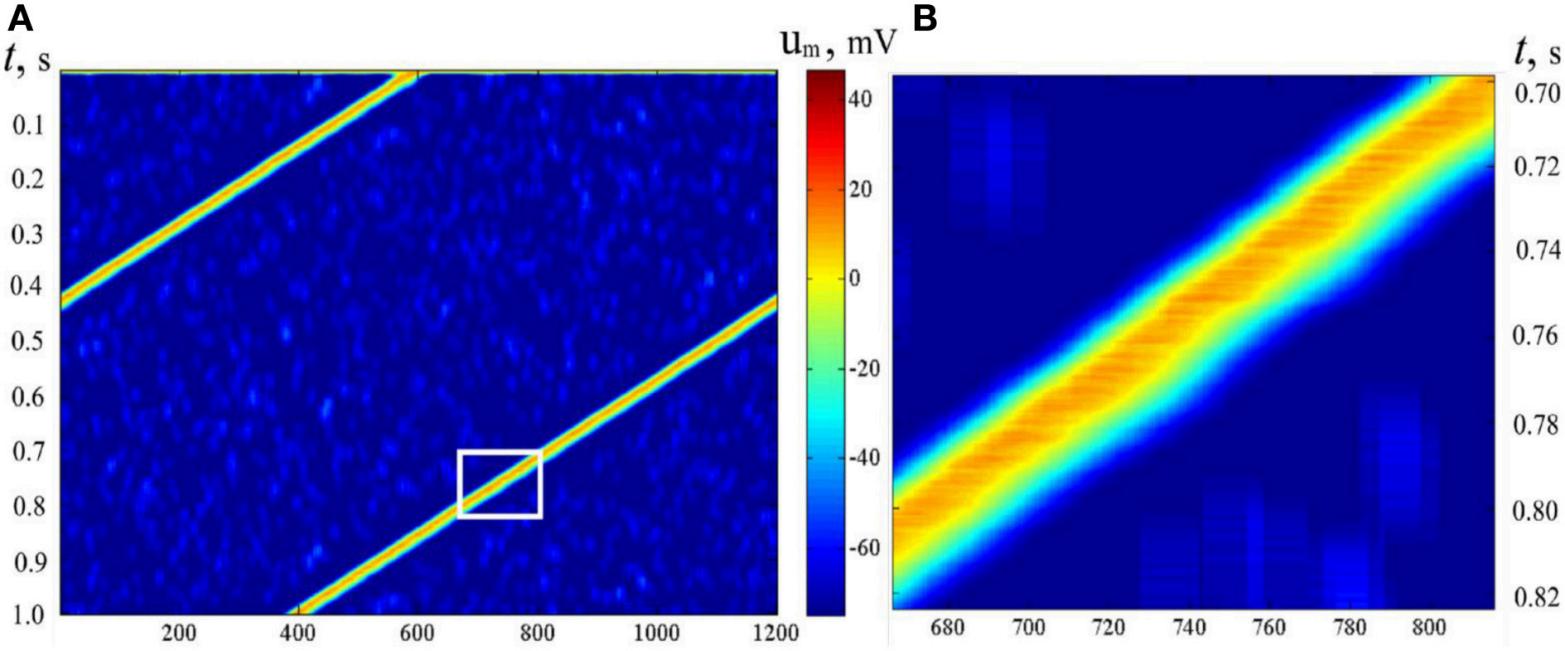
**Activity propagation in LIF dynamic neural network**. Notations as in Figure 11. **(B)** presents the rectangular inlet of **(A)**. Asymmetric excitatory connections between neurons: from neurons with larger marker order numbers backwards. *N* = 600, *M* = 1200, *L* = 30. The figure gives an example of a wave, which moves over the states of activity of the neural network with asymmetric connections.

In Sections Pre-formed Attractors, *d* = 1 (pre-formed bump attractors) and SOM Type Learning in Static Bump Attractors we studied the structure of neural attractors with help of auxiliary dynamics, adding accommodation to neural properties. The dynamical properties of the neural networks can be set by appropriate learning (Dunin-Barkowski, 1984, 1986; Dunin-Barkowski and Osovets, 1995) or by forming of the connections (Stringer et al., 2002). The dynamical properties of the network can be also obtained with the help of molecular markers, if connection forming rules are asymmetric. In simulations, connection rules in the network were analogous to the connection rules in Section Pre-formed Attractors, *d* = 1. However, connected with excitatory connections were made only from neurons with larger order numbers of markers to the neurons with smaller order numbers, up to number difference of *L*/2. Figure 12 demonstrates activity of such a type of neural network. The period of the activity in this case depends on threshold control and can be varied in some range [the early example of such a control is given in Figure 1 of Dunin-Barkowski (1986)].

It should be emphasized that the pattern of the activity of the neural network in Figure 12 gives a clear picture of propagation of a wave in excitable media (Wiener and Rosenblueth, 1946). In Figure 12B one can see that the excitation wave form is asymmetric. Note that the upper-left edge of the excited area at Figure 12B (the front edge of excitation wave) looks more straight and sharp than the lower-right edge (the tail of the wave), alike, say, a wave of flame in the field of dry grass. However, in this case, physically, there is no excitable media. The wave propagates, in fact, in the configurational space of the neural network. The existence of such kind of waves was also observed in (Dunin-Barkowski, 1984).

## Discussions

### Dimensions

Continuing the ideas of Section Preformed Attractors, *d* = 1, Visualization of Ring Attractors it is worth to make a note on properties of attractor networks with *d* > 1. In particular, it can be argued that the dimensionality of a reliable attractor neural network with a good resolution is hardly possible for *d* > 4; in any case, it can be shown that it cannot exceed the value *d* = 8.

Let us have a preformed continuous bump attractor of dimensionality *d* with *d*-cube grid, constructed with of *d*-dimensional analogs of the molecular-marker method (see Section Pre-formed Attractors, *d* = 1). In this case, each neuron gets *k* markers. With help of each marker, the neuron is connected with (2δ)^*d*^ neurons (the volume of the *d*-dimensional neuronal neighborhood). So, each neuron has (2δ)^*d*^ · *k* connections. This number must be less than the number of neurons: (2δ)^*d*^ · *k* < *N*. Let *l* be a number of discrete elements for each dimension. Each element should be presented as a separate attractor state of the network. So, we have *l*^*d*^ ≤ *N*^2^∕*L*, or *d* ≤ log(*N*^2^∕*L*)∕log(*l*). The estimate of maximal value of *d* can be obtained in the following way. First, note that this estimate at fixed *N* increases with the decrease of *L* and *l*. For the number of neurons in the range *N* = 10000÷50000 (the usually supposed number of neurons in one cortical column in human brain), we get *d* = 7÷8 for *l* = 10, *L* = 10 and *d* = 3÷4 for *l* = 100, *L* = 100. In this paper, we have studied only the cases of *d* = 0 and 1.

### Two layers perceptron implemented with isolated points attractor

In Sections Molecular Marker-based Attractors, *d* = 0 and Robustness to Noise, *d* = 0 we have introduced and explored neural networks with inborn point attractors (*d* = 0). Properties of these attractor neural networks are similar to properties of Hopfield networks. We have revealed the functionally important properties of these attractors. When we have constructed a perceptron, i.e., the neural network, which detects specific patterns, it occurred that attractor-based perceptrons substantially surpass neuron-based perceptron's error tolerance (see Figure 3B).

### Molecular marker based neural network with 1-*d* bump attractor

In this paper we have developed a physiologically plausible mechanism to make connections in the network to have in it a “long” one-dimensional attractor, i.e., the attractor with the number of states, exceeding the number of neurons in the network. We have demonstrated examples of such networks, obtained in computational experiments. Our examples can serve as templates for interpretation of neurophysiological experiments, as one-dimensional neuron attractors are often supposed to be present in different brain structures. For the experimental verification purposes we have proposed two methods for visualization of neural attractor activity. The methods were tested on data of computational experiments and might be used for physiological experiments.

### Extension of kohonen's SOM

We explored the bump attractor-based SOM, similar to Kohonen's original construction [preliminary results were published earlier (Solovyeva, 2013)]. The construction demonstrates a series of considerable distinctions from the original Kohonen's paradigm. First of all, it is more likely to be implemented in a real brain than the original construction of Kohonen, as Kohonen's chains and grids (Kohonen, 1982) have no analogs in real neural systems. Second, the neural activity regenerative processes in bump attractor network provides the natural neighborhood of the attractor states by the mere nature of the bump attractor. Indeed the neighborhood of a given state consists of the states, which are close to a given state by Hamming metrics. In the Kohonen's SOM, the neighborhoods of the nodes are defined forcefully.

Thus, computational experiments on the learning of neural networks with bump attractors to respond to external signals have demonstrated that SOM-like mechanisms can be efficient for representation of continuous variables in realistic neuronal systems.

### Neural building blocks

It was believed for a long time that neural information processing and neural control is based on a set of “principles of neural organization,” just as the artificial information and control machines use standard operations and standard circuits (Hebb, 1949; Brindley, 1969; Dunin-Barkowski, 1971a,b; Ito, 2012). There is a hope that the number of such concrete circuit principles is, although large, is not huge (say, < 1000). One of the ways to complete the “brain reverse engineering” (Stevens, 1985) is to reveal these principles, one by one, in order to obtain a complete set of them (Dunin-Barkowski, 2011b). The bump attractor neural structures (with *d* = 0 and 1) and their versions definitely constitute a part of this set of principles (Knierim and Zhang, 2012). In this paper, we have presented only fragments of the future detailed description of the ways of functioning and functions of bump attractors. There is still a long way to go until discovery of new principles and for this knowledge to be applied in future artificial mind systems.

### The clock-brain machinery

We hope that hereby we have demonstrated that the simple operation of switching on of stable static or dynamic recurrent states, provided by inborn neuronal connections, can serve as a basic computational operation of the neural systems in many cases. In this capacity, the equal utility might be assumed for the neural networks with attractor dimensions *d* = 0, 1, …, ≤ 8. However, it is possible that the case of *d* = 1 is in fact special and might be considered a basis for a large class of interdependent constructions which enable the effective functioning of the neuronal systems. On one hand, such a conclusion might be based on the fact that multi-dimensionality can be often considered a Cartesian product of the appropriate number of one-dimensional constructions. On the other hand, there emerges a new class of structures in neuronal systems that is based on the networks with *d* = 1. We would propose to name this class “The Clock-Brain Machinery.” What does this mean? It would be relevant to recollect the remark of Masao Ito (Ito, 2012) that states that the purpose of understanding concrete human-made mechanisms in the long history of their existence in human culture has been, in essence, for disassembling and further successful reassembling of these mechanisms. For centuries, the most sophisticated of them were the mechanical clocks. Ito expresses the opinion that the likely considerations should be used for analysis of the brain machinery. This note might be treated as allegory, but there are arguably more concrete elements in it as well. We suppose that the dynamic ring attractor neural network (Figure 12) can serve as a basic block of the realistic clock metaphor. One should also bear in mind that static attractor one-dimensional structures can be easily transformed into dynamic one-dimensional attractor structures (Dunin-Barkowski and Osovets, 1995; Hopfield, 2010) with switching on of the Ca++-dependent potassium channels. The complete cycle of activity in the network of the type of Figure 12 resembles the full turn of a clock's gear wheel. The activity of different cyclic networks can be easily connected, yielding a structure resembling the gear-wheeled clock mechanism. Of course, it should be taken into consideration that specific states of neural “gear wheels” can have different meanings. Of course, the clock-wise picture of the brain machinery doesn't pretend to present all of a real brain's processes, but it might yield a convenient framework for getting more details of the brain function.

## Conclusion

In this paper we extend our work on attractor based neural networks. Special attention is paid here to aspects of neural dynamics insufficiently highlighted beforehand. In this paper, we have demonstrated that robustly functioning neural networks of *N* neurons can have *M* = *k*·*N* attractor states, where *k* = 1÷1000. On the other hand, there is a possibility, that the value of *k* as low as 10^−2^–10^−4^. For example, it is believed that the typical “grandma neuron” is definitely a representative of a sub-network, containing $N_{\text{grandma}}=10^{2}-10^{4}$ neurons (Quiroga et al., 2013). The latter assumption cannot be overturned. If the neurons, which take part in one “grandma” representation are not used for other purposes, then we get the very low estimate of *k* = 1∕*N*_grandma_. As a result, one of the main problems in experimental and theoretical neural studies is to understand if neuronal elements in brain are used to represent only unique external events, or if they can be used in combinations, so that different combinations of active neurons can represent different external events. The fact of the existence of remapping in the hippocampus (Fyhn et al., 2007) shows that at least in some neural structures, the combinations are used. For the attractor neural networks, we have considered in a general form the networks whose set of attractor states might represent finite grids for discrete and continuous variables. This treating naturally leads us to notions of neural networks with attractor dimensions *d* = 0, 1, 2, etc. We give simple calculations, showing that for biological neural networks *d* is (fussy) limited, *d* =≤ ~8.

Further, we consider a method (“molecular markers”) for forming inborn connections in neural networks, which provide neural networks with attractor dimensions *d* = 0.1. The *k*-value in the cases we have considered is in the range *k* = 1÷1000. The elaboration of inborn neural networks with attractor dimensions *d* > 1 is an interesting problem for future works. Also, we note that the activity of neural networks with inborn attractors can obtain meaning due to inborn or learned forming of connections of a concrete neural network with other neural structures. In pursuing this idea, we showed our results for the cases of *d* = 0 (two-layer perceptrons) and *d* = 1 (Kohonen's SOM, based on one-dimensional attractor states). The last part of our paper demonstrates that the results, obtained with McCulloch–Pitts neural model, have direct analogies to the networks of impulse neurons of the LIF type. There is no reason to assume that it doesn't hold true for more detailed physiological neural models. This model may find applications in many areas including deep neural nets for image, text and voice recognition, autonomous driving and drug discovery and drug repurposing and provide a theoretical base for further research in applying biological principles to machine learning.

### Conflict of interest statement

The authors declare that the research was conducted in the absence of any commercial or financial relationships that could be construed as a potential conflict of interest.
