# Supplementary material for: Models of Innate Neural Attractors and Their Applications for Neural Information Processing
Source: Front Syst Neurosci. 2016 Jan 5;9:178. doi: 10.3389/fnsys.2015.00178 (PMC4700189; doi:10.3389/fnsys.2015.00178)
Supplement: Supplementary file 1 [file Presentation1.pdf]

Supplementary Material 1. The neural models

We use several types of neural models in calculations. Hereby the main features of the models are described in detail.

A1.1. McCulloch-Pitts (MCP) neural model.

In this case, the behavior of the *i*-th $(i = 1, ..., N)$ neuron is described by its phase function, $\varphi_i(t)$. There are two constants: the duration of excitation, *w*, and the duration of refractoriness, *r*. If $\varphi_i(t) \geq r$ and $(\sum_{j=1}^{N}(w_{ji} \cdot x_j(t)) - \theta_i) \geq 0$, then $\varphi_i(t+1) = 0$, otherwise $\varphi_i(t+1) = \varphi_i(t) + 1$. The output of the neuron $x_i(t) = 1$, if $\varphi_i(t) < w$, otherwise $x_i(t) = 0$. Usually, $w = 1$ and $r = 0$.

The important rule for the MCP neural networks is the rule of updating the values of $\phi_i(t)$ and $x_i(t)$. The rule, described above, is known as synchronous dynamics: the phases of all neurons are updated simultaneously. Sometimes, we used the asynchronous random dynamics. In this case, the updating is performed in cycles of *N* updates. In each cycle of updates, the order of neurons is selected randomly and, in this order, neurons are updated one by one; the freshly updated neuron takes part in updating the next neurons in that cycle of *N* updates.

To explore the set of equilibrium states of the neural networks we introduce the accommodation mechanism into neural network dynamics (Dunin-Barkowski, Osovets, 1995). With this purpose in mind we accept that in moments, when neuron switches into the excited state, its threshold gets increases of a constant value $\Delta\theta$, while it decreases all the time exponentially with the time constant τ toward the final value of θ_0:

$$\theta(t) = (\theta + \Delta\theta \cdot \partial(t\text{-}t_i) - \theta_0)e^{-\frac{t}{\tau}} + \theta_0,$$

Where t_i is the moment of the *i*-th transition of the neuron into the excited state and

$$\partial(t\text{-}t_i) = \begin{cases} 1, \; npu \; t = t_i \\ 0, \; npu \; t \neq t_i \end{cases}.$$

Thus, when the neural network stays for a long time in a fixed stationary state, the Hopfield energy of this state increases and the network activity moves to the adjacent state with lower energy.

A1.2. LIF model

The dynamics of these neurons is described by the following equation

$$\tau_m \frac{du_m(t)}{dt} = -\left(u_m(t) - u_r\right) + R_m\left(i_{syn}(t) - i_{inh}(t) - i_{Ca}(t)\right) \qquad \text{(A1.1)}$$

with

$$\tau_{Ca} \frac{di_{Ca}(t)}{dt} = -i_{Ca}(t), \qquad \tau_{Ca} = 0.1 \text{ s} \qquad \text{(A1.2)}$$

Here, $u_m(t)$ - membrane potential, $\tau_m = R_m C_m$ - membrane time constant, $C_m = 1$ nF and $R_m = 10$ MΩ - capacitance and resistance of the membrane, $u_r = 0$ mV - resting potential of the membrane, $i_{syn}(t)$ - the sum of excitatory synaptic currents, $i_{inh}(t)$ - the inhibitory current, $i_{Ca}(t)$ - the accommodation current, which depends on activity of neurons. This current is considered to reflect the action of Ca^{++}-dependent K^+ channels. With each spike $i_{Ca}(t)$ gets an increase of 1 nA. The neuron generates an impulse (spike) when its membrane potential $u_m(t)$ attains threshold u_{th}. Afterwards, its membrane potential momentarily jumps to zero value, while threshold gets increase of 1000 mV and then decreases according to equation:

$$\tau_{th}\frac{du_{th}(t)}{dt}=-\left(u_{th}(t)-u_{th0}\right) \qquad \text{(A1.3)}$$

with $\tau_{th}=2\text{ ms}$, and $u_{th0}=10\text{ mV}$. Each incoming impulse momentarily adds 0.3 nA to $i_{syn}(t)$. In the absence of incoming impulses, $i_{syn}(t)$ decays exponentially to zero with time constant of 25 ms.

The inhibitory current, $i_{inh}(t)$, is the same for all excitatory neurons in the network and represents a global variable which controls the activity of the network. It is controlled by the following equations:

$$\tau_e\frac{di_e(t)}{dt}=-i_e(t)+N\sum_j\sum_f\delta(t-t_j^f) \qquad \text{(A1.4)}$$

$$\begin{cases} i_{inh}=k_{inh}\left(i_e(t)-I_{e0}\right), & if \quad i_e(t)>I_{e0}, \\ i_{inh}=0 & otherwise \end{cases}, \qquad \text{(A1.5)}$$

Variable $i_e(t)$ represents the excitatory input to inhibitory neurons, $\tau_e=10\text{ ms}$, $k_{inh}=const$, $I_{e0}=10\text{ nA}$. The synaptic delays between neurons were uniformly randomly distributed in the range 1.0 – 5.0 ms.

Supplementary Material 2. Number of inborn attractor states for d=0

The mean value of matrix elements is:

$$\gamma=\overline{T_{ij}}=\Pr\{T_{ij}=1\}=1-\Pr\{T_{ij}=0\}=1-\left(1-\frac{L^2}{N^2}\right)^M. \qquad \text{(A2.1)}$$

We suppose that M is proportional to N^2/L^2, i.e. $M=\kappa\cdot(N^2/L^2)$. When $M\to\infty$, we have:

$$\gamma=1-\left(1-\frac{\kappa}{M}\right)^M=1-e^{-\kappa}. \qquad \text{(A2.2)}$$

Now, let the network have at the input one of its theoretical attractor patterns. Then, the probability, that a ‘foreign’ neuron have L excitatory inputs is

$$P\{h_i=L\}=\gamma^L.$$

So, the probability that at least one of (N-L) foreign neurons will get L units of excitation is:

$$P_{err}=P\{\exists i_{inactive}:\ h_i=L\}=1-(1-\gamma^L)^{N-L}\approx 1-(1-\gamma^L)^N$$

Taking $P_{err}=1/N$, we have:

$$1-(1-\gamma^L)^N=\frac{1}{N},$$

and, after non-complicated transformations, leaving only first order (by 1/N) terms, we have:

$$\gamma=N^{-2/L}. \qquad \text{(A2.3)}$$

Comparison of (A2.2) and (A2.3) finally yields:

$$\kappa=-\ln\left(1-N^{-2/L}\right). \qquad \text{(A2.4)}$$

Table A2 gives numerical values of κ for a set of N values at L=20. Although computational estimates of κ give the constant value $\kappa=1$, one can see that the analytical reasoning does not diverge too far from the computational experiments estimate. Thus, Table A2 shows that theoretical estimates of κ for $N\le 5000$ are less than two times smaller than 1.0.

N	100	500	1000	2000	3000	500
κ	1.0	0.77	0.69	0.63	0.6	0.56

Supplementary Material 3. Distances between states in 1 *d* bump attractor obtained with help of model molecular markers

Fig. A1 presents a fragment of the layout of all neurons in order of the order numbers of the markers, which they contain (as each neuron has k markers, each neuron is presented *k* times in this layout). The neurons become excited in the same order, while the activity propagates over the bump attractor. We now consider the distance between some initial state, X_0 and states which are following it, $X_1, X_2, X_3, ... X_t$, (*t*=1, 2, … *M*).

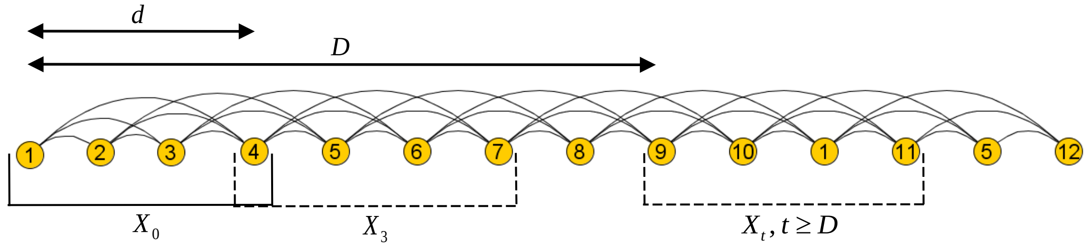

Fig. A1

Fig. A2 shows the inner product (X_0, X_t) of two states plotted against t. It is obvious that in the beginning, the intersection between X_0 and X_t decreases linearly from *L* to 0. Then, it stays 0 up to $t = D$. For $t > D$, the intersection is random. Its mean value can be obtained as follows. Each neuron of the first state takes later part in (*k*-1) states That means that for any neuron which is excited in X_0, the probability to be excited in X_t (for large *t*) is

$$p = \frac{(k-1)L}{M-L}.$$

That means that the average intersection between X_0 and X_t will be $pL \approx (k-1)L^2 / M$

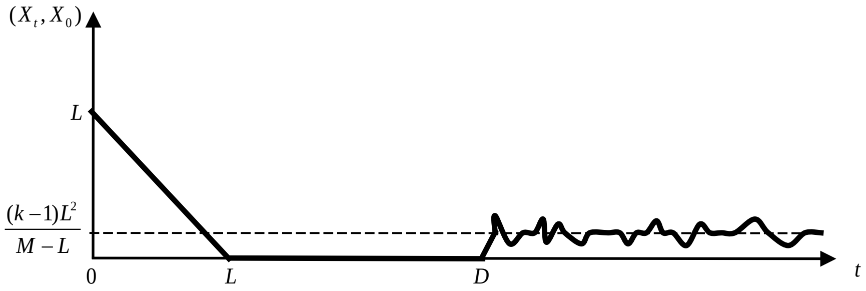

Fig. A2

The Hamming distance between the states is connected with intersection of them according to the relation $H(X_t, X_0) = 2(L - (X_t, X_0))$, where from we get for $t \geq D$

$$\underline{D} \approx 2L\left(1 - \frac{(k-1)L}{M}\right).$$

With *L*=15, *k*=3, *M*=900, we have $\underline{D} \approx 29$, which coincides with the results of the computational experiments.

Supplementary Material 4. Evaluation of k_c in one-dimensional bump attractors

As each neuron takes part in *k* attractor states, each line of the matrix *T* contains about $2\delta k$ positive (equal to 1) matrix elements. The probability of positive matrix element is $2\delta k / N$. That means that the probability of firing of one excessive neuron, which is not active in X_0, is $(2\delta k / N)^L$. On the contrary, the probability that this will not happen for any of the remaining $N - L$ neurons is $\left[1-(2\delta k / N)^L\right]^{N-L}$.

In computational experiments, the critical value k_c was defined as the value of *k*, such that five random networks with a given *k* show perfect cycles. Thus, for k_c we obtain an equation:

$$1-\left[1-\left(\frac{2\delta k_c}{N}\right)^L\right]^{N-L} < \frac{1}{5}$$

And finally, for the critical value of *k*, we have:

$$k_c < \frac{N}{2\delta \sqrt[L]{5N}}, \qquad \text{(A4.1)}$$

Factor $\sqrt[L]{5N}$ is of the order of 1 and changes very slowly with *N*. So, (A4.1) yields practically linear dependence of k_c on *N*. (cf. Fig. 17). In particular, we have:

at L = 15, N = 300, $\sqrt[15]{5 \cdot 300} \approx 1.62$, k = 7.7 (in experiment, k = 5);

at L = 15, N = 1200, $\sqrt[15]{5 \cdot 1200} \approx 1.79$, k = 28 (in experiment, k = 21).

Supplementary Material 5 Trade-off relations for network attractors

This section uses the computational experiments first presented to the conference "Neuroinformatics 2013" (Karandashev, 2013). For neural network applications, it is important to know how the network behaves depending on the values of its parameters. In particular, it is important to know how stable the attractor points are. Fig. A3 shows the "error" in states, as a function of *N*, *M* at $L = 20$, obtained in Monte Carlo computational experiments. The synchronous *L* winners dynamics (Supplementary Material 1) of the network was used and the "error" was considered to be the Hamming distance between the experimentally obtained stable state and the "theoretical" attractor point which served as the initial condition. It can be seen that the error grows with *M*. For *M* less than a critical value, M_{cr}, the "theoretical" attractor points are stable.

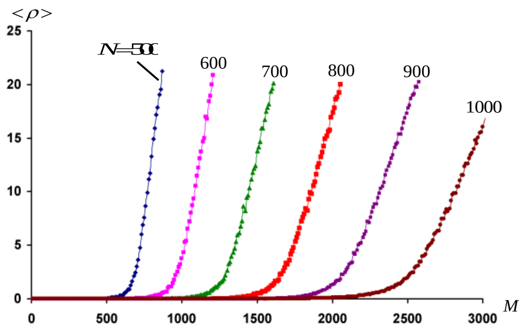

Fig. A3. The “error” in neural network stable states

N and M are the number of neurons and the number of marker types. Each type of markers has the same number, L=20, of elements. In each case, plotted are values averaged over all M “theoretical” attractor points.

Fig. A4 shows dependence of $\alpha_{cr} = M_{cr} / N$ on N. The linear empirical approximation shows that with increasing N, the value of M_{cr} increases $\approx N^2$ (when L is constant): $M_{cr} \approx (N / L)^2$.

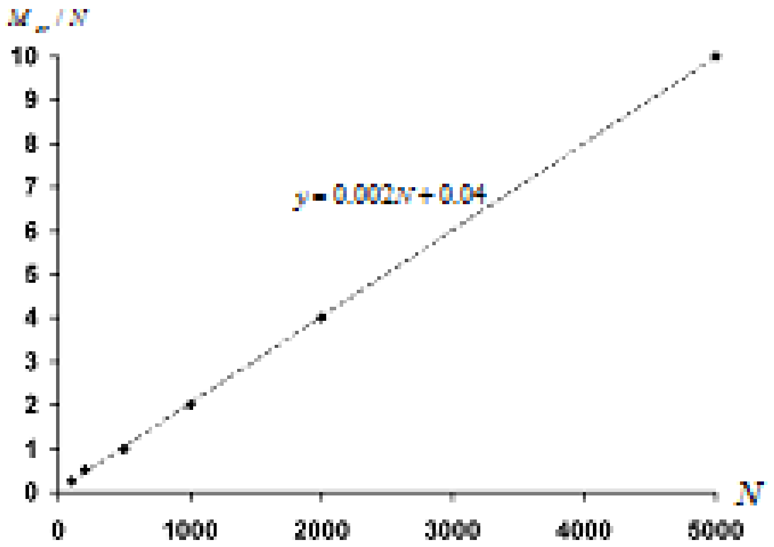

Fig. A4. The critical values of ratio M_{cr} / N as a function of N

Computational experiments (dots); broken line is the least square regression. L=20.

The mean value of matrix elements is

$$\overline{T_{ij}} \cong ML^2 / N^2 \qquad (A5.1)$$

When network resides at attractor state, S^m, the L neurons which are active get the following inputs:

$$h_{act} = \sum_{j=1, j\neq i}^{N} \mathbf{T}_{ij} S_j^{\ m} = L - 1 \qquad (A5.2)$$

Input to the rest ($N - L$) of the neurons is approximately:

$$h_{inact} = L \cdot \overline{\mathbf{T}_{ij}} \qquad (A5.3)$$

Here $\overline{\mathbf{T}_{ij}}$ is the average value of matrix element of $\mathbf{T}$. The distinction between the right parts of (A5.2) and (A5.3) enables the neural network discriminate between attractor and non-attractor states. Supplementary Material 2 gives the analytical reasoning, which qualitatively explain the data of computational experiments, displayed in Figs. A3 and A4.

It is known that not all sets of points can be separated by a plane into two subsets. For example, any four points in 3-d space can be divided by a plane in any combinations, but for five points that can not always be done.

A general result related to this problem has been obtained by E. Gardner (Gardner, 1988). It states that if there are randomly chosen 2*R* points in the R-dimensional space, painted randomly in two colors, *R* points in each group, then at $R \to \infty$, with probability approaching 1, exists the plane which separates the colored points by colors. But when the number of points exceeds 2*R*, the probability of separation approaches 0 as $R \to \infty$. This result states that at maximum, we can separate only 2*R* points in *R*-dimensional space. In the case of separating a few points from the others, the situation changes. Let us have M points in R-dimensional space. Let then divide them into two parts: k points in one of them and $M-k$ in the other. In this case (formula (40) of (Gardner, 1988)):

$$\alpha_c = -\frac{1}{(1-m)\cdot\ln(1-m)} .$$

Where, in our case, α_c and m are correspondingly $\alpha_c = M/R$ and $m = 1-2k/M$. So the expression for relations between *R*, *M*, and *k* is:

$$R = 2k\cdot\ln(M/2k) \qquad \text{(A5.4)}$$

We tested this theoretical estimation in computational experiments for a set of different values of *k* and *R* (Fig. A5):

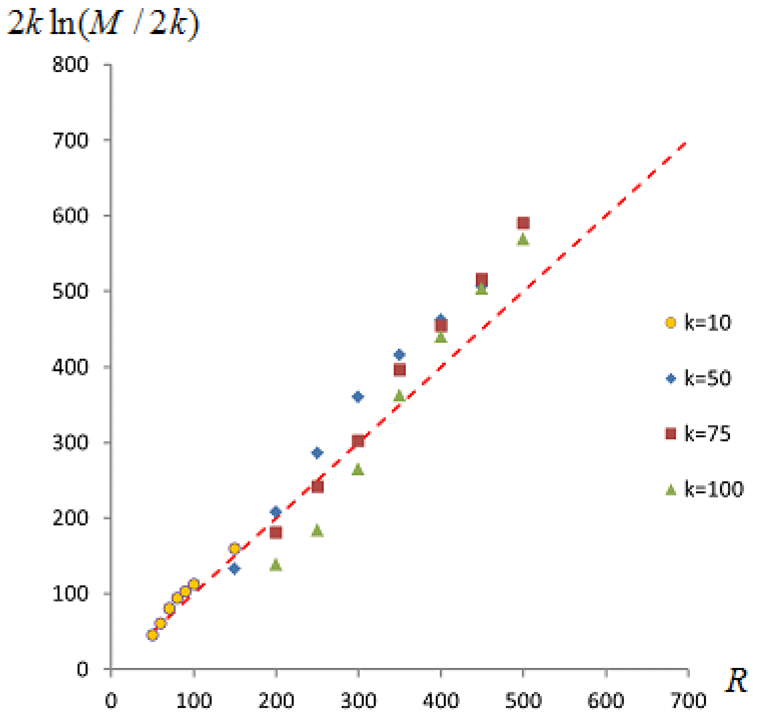

Fig. A5. Data of computational experiment on relation between *M*, *R* and *k*

The linear separation was performed with the help of Rosenblatt algorithm. The broken line corresponds to equation (A5.4). Parameters are shown in the figure.

So, the number of patterns M when k is fixed is an exponent of R. Further, we use $k = (ML / N) \approx 0.01M$, and R in the range 100 – 500.
